# Hyoid displacement patterns in healthy swallowing

**DOI:** 10.31744/einstein_journal/2022AO6268

**Published:** 2022-03-04

**Authors:** Maria Regina Macedo Alves, Isabella Christina Oliveira, Guilherme Maia Zica, Henrique Lederman, Maria Inês Rebelo Gonçalves

**Affiliations:** 1 Universidade Federal de São Paulo São Paulo SP Brazil Universidade Federal de São Paulo, São Paulo, SP, Brazil.

**Keywords:** Deglutition/physiology, Hyoid bone/anatomy & histology, Hyoid bone/physiology, Aged, Videofluoroscopy

## Abstract

**Objective:**

To describe the patterns of displacement of the hyoid bone in healthy individuals, considering their displacements during swallowing of different consistencies.

**Methods:**

Two hundred one swallowing videofluoroscopy exams of 67 adult and elderly individuals without swallowing disorders were analyzed. Descriptive analysis was performed to identify and describe the patterns of displacement of the hyoid bone.

**Results:**

Seven types of displacement of the hyoid bone were found: H1 (horizontal), H2 (short vertical and long horizontal), H3 (vertical and diagonal to upper), H4 (long vertical and short horizontal), H5 (vertical), H6 (diagonal), and H7 (brief). The standards were maintained in different consistencies. The most frequent pattern of displacement was type H2. The distribution of the types of displacement of the hyoid was different among men and women.

**Conclusion:**

Seven patterns of displacement of the hyoid bone during swallowing of normal adults and older people have been described. The most frequent pattern of displacement was horizontal, with variations in distribution between men and women. The displacement pattern was maintained during the swallowing of the three different consistencies (thin, pasty and solid liquid).

## INTRODUCTION

Swallowing is a complex neuromotor process that involves the facial, oral, pharyngeal, laryngeal, cervical and esophageal muscles. This process can be divided into three phases: oral, pharyngeal, and esophageal.^(1,2)^ Among the several movements of the structures involved in swallowing, the displacement of the hyolaryngeal complex stands out as an important mechanism in this process.^(3,4)^

Studies in the literature consider that the hyoid bone, together with the laryngeal complex, moves forward and upward, with the vertical movement primarily associated with laryngeal vestibule closure, and the horizontal movement mainly associated with cricopharyngeal muscle traction.^(3,5)^ This mechanism allows the upper esophageal sphincter to open and the epiglottis to tilt, therefore, favoring bolus transit.^(6-8)^

The hyoid bone helps anchor the supra- and infrahyoid muscle groups and supports the laryngopharyngeal region. Contraction of the suprahyoid muscles causes the hyoid bone to move upward and forward. Such displacement, together with the tongue during bolus ejection and subsequent contact with the hard palate, ensures laryngeal elevation and anteriorization with adequate force and duration.^(2-4,9)^ This biodynamic favors lower airway protection, preventing supraglottic penetration and laryngotracheal aspiration of saliva and/or food.^(10)^

Ekberg,^(11)^ by analyzing 50 healthy subjects submitted to videofluoroscopy of swallowing, reported that the hyoid bone movement is precise and has little variability. The movement goes up and forward in a straight line from its resting position to its maximum elevation, and questioned the clinicians’ conception that the hyoid bone performs a curved and variable path. However, clinical trials^(3,4,12-15)^ have described significant variations in swallowing from non-dysfunctional subjects. Thus, the theory arose that the movement of the hyoid bone may be more individual than what is traditionally believed.

Senecail et al., by analyzing anatomical sections, radiographs, and videofluoroscopy described variable movements of the hyoid bone during speech articulation and stated that its displacement varies according to the type of phoneme.^(16)^ This information led to the assumption that, as in speech, the hyoid bone could also present variable displacements during swallowing. Considering this hypothesis, we have chosen to study videofluoroscopy exams, as this resource allows a detailed analysis of the feeding process in all its phases.^(4,17)^ Several authors have described the displacement of the hyoid bone in the vertical and anterior directions.^(3,12-15,18-20)^ Studies suggest that the amplitude of hyoid bone displacement in the healthy population is highly variable, however, the aspects and characteristics of its movement remain poorly explored.^(3,21)^

In the last 5 years, there has been a concern for the analysis of swallowing dynamics with little specificity in the evaluation of hyoid bone displacement in different types of pathology. To date, there are no descriptive studies evaluating the different patterns of hyoid bone displacement and their variations during swallowing in individuals without dysfunctions.

## OBJECTIVE

To describe the displacement patterns of the hyoid bone in healthy subjects, considering their movements during swallowing of different consistencies.

## METHODS

The study was approved by the Research Ethics Committee of the *Universidade Federal de São Paulo* (UNIFESP), declaration # 758.172, CAAE: 34302914.0.0000.5505. All participants signed the consent form.

This study was carried out by means of the analysis of objective swallowing exams from records of the Functional Rehabilitation of Swallowing Sector of the Speech Therapy Department of UNIFESP. The inclusion criteria were individuals aged ≥18 years of age and with a diagnosis of normal swallowing after an objective examination of swallowing. We excluded individuals any degree and type of dysphagia after the objective examination of swallowing. The classification of the swallowing disorder was done by means of the clinical findings from the videofluoroscopy by two speech therapists with experience in the area and, in case of disagreement, a third evaluator was called.

At the institution’s database, from January 2014 to December 2016, 67 exams of adult and older individuals with reports of normal swallowing were found.

The videofluoroscopic swallowing exams were performed using a Siemens Axiom X-ray machine (500mA, 150kv automatic, 30 frames/second) with seriograph and closed circuit recording. The videos were edited in CyberLink^®^ PowerDVD9 software for frame-by-frame analysis and screen image capture. Patients were examined in left lateral orthostatic position, at rest and during swallowing of thin (30mL of mineral water and 10mL of 100% barium sulfate gel), pasty (5mL in a spoon, one third of 100% barium sulfate gel in two thirds of petit suisse yogurt), and solid (one quarter of cheese bread soaked in 100% barium sulfate gel) liquids.^(9)^

At the time of the radiological examination, all patients had exclusive oral feeding, hydration and no history of pneumonia. After identifying the exams on record, we performed a qualitative visual analysis of the images in lateral view. We established the hard palate as the upper limit, the neck as the anterior limit, the larynx as the lower limit, and the cervical spine as the posterior limit. The displacement of the hyoid bone was evaluated and described by 201 videos by two speech therapists with experience in the area, in the agreement form. If the description was inconsistent, a third rater was required to break the tie. We chose not to carry out statistical analyses, since the objective of the study was not to standardize or prove correlations. A descriptive analysis was carried out, subdividing the types of displacement found, aiming to identify the patterns of displacement and understand the complexity and diversity of this displacement in the swallowing of healthy individuals.

## RESULTS

Sixty-seven individuals were evaluated, 36 of them were women (53.74%). The mean age was 51.5 years (±16.97) with a median of 51 years, ranging from 21 to 93 years.

In the female group, the mean age was 47.72 years (±15.99) with a median of 48 years, ranging from 21 to 81 years. In the men group, the mean age was 55.9 years (±17.27), with a minimum of 25 and a maximum of 93 years, and a median of 60 years.

The group was composed of 41 adults (<60 years; 61.2%), aged 21 to 59 years (mean 40.63±10.98 years and median 41 years), and 26 elderly (≥60 years; 38.8%), aged 60 to 93 years (mean 68.65±8.24 years and median 67.5 years).

Seven types of hyoid bone displacement were found, with the qualitative findings described in table 1 and the distribution in table 2. It was observed that the pattern belonging to the individual was repeated during the ingestion of all consistencies (thin liquid, pasty and solid), so that no patient had more than one displacement pattern.

**Table 1:** Description of the types of hyoid bone displacement during normal swallowing found by means of the swallowing videofluoroscopy findings in 67 subjects

Type	Displacement	Anteriorization and elevation relationship	Graphical representation
H1	Horizontal	Only anteriorization	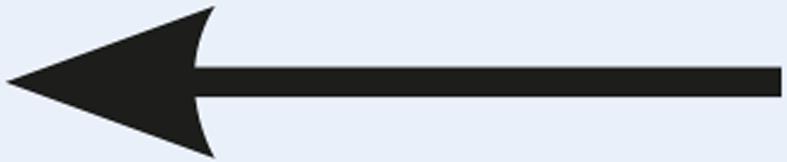
H2	Short vertical and long horizontal	Anteriorization predominates over elevation	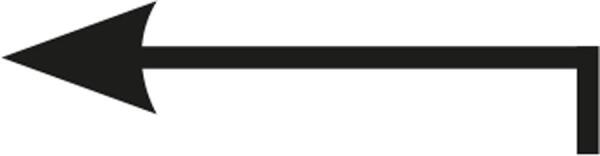
H3	Vertical and diagonal to top	Anteriorization to diagonal predominates in relation to elevation	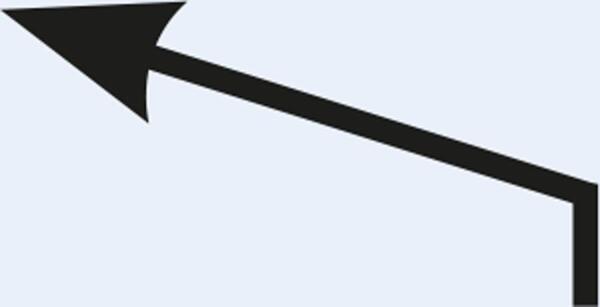
H4	Long vertical and short horizontal	Elevation predominates with respect to anteriorization	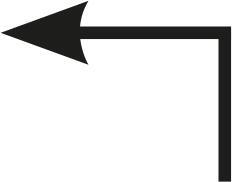
H5	Vertical	Only displacement in the vertical (superior) plane	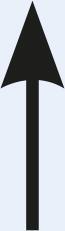
H6	Diagonal	Elevation and anteriorization without specific predominance	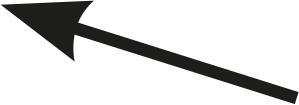
H7	Short	Short elevation and anteriorization	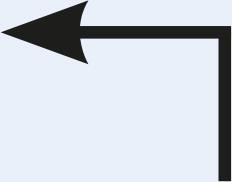

H1: horizontal; H2: short vertical and long horizontal; H3: vertical and diagonal to top; H4: long vertical and short horizontal; H5: vertical; H6: diagonal; H7: short.

**Table 2:** Distribution of the total sample (n=67), regarding the type of hyoid bone displacement

Displacement type	
H1	12 (17.91)
H2	39 (58.20)
H3	1 (1.49)
H4	1 (1.49)
H5	1 (1.49)
H6	2 (2.98)
H7	11 (16.41)
Total	67 (100)

Results expressed by n (%).

H1: horizontal; H2: short vertical and long horizontal; H3: vertical and diagonal to top; H4: long vertical and short horizontal; H5: vertical; H6: diagonal; H7: short.

The most prevalent hyoid bone displacement pattern showed predominance of anteriorization over elevation, the H2, with 39 individuals, corresponding to 58.20% of the sample. This was followed by 12 individuals with only an anteriorization pattern (H1), representing almost 20% of the group. Short elevation and anteriorization (H7) was described in 11 individuals (16.41%). The dislocations H3, H4, H5 and H6 were the ones with the lowest appearance in the objective swallowing examination, with less than 3% in all categories (Table 2).

A similar distribution was observed considering the types of hyoid bone displacement and age group. The total percentage value of patients with H2 displacement was 56.10% in adults and 61.53% in the elderly. Displacement was the most prevalent in elderly group, followed by H1 and H7 (Table 3).

**Table 3:** Distribution of adult and elderly women and men, regarding the type of hyoid bone displacement (n=67)

Type	Adults (<60 years)			Older people (≥60 years)		
	
	Women	Men	Total	Women	Men	Total
H1	8 (30.80)		8 (19.51)	4 (40.00)		4 (15.40)
H2	14 (53.80)	9 (60.00)	23 (56.10)	6 (60.00)	10 (62.50)	16 (61.53)
H3	1 (3.80)		1 (2.44)			
H4	1 (3.80)		1 (2.44)			
H5					1 (6.30)	1 (3.84)
H6		2 (13.30)	2 (4.88)			
H7	2 (7.70)	4 (26.70)	6 (14.63)		5 (31.30)	5 (19.23)
Total	26 (100)	15 (100.00)	41 (100.00)	10 (100.00)	16 (100.00)	26 (100.00)

Results expressed in percentages (%).

H1: horizontal; H2: short vertical and long horizontal; H3: vertical and diagonal to top; H4: long vertical and short horizontal; H5: vertical; H6: diagonal; H7: short.

Although the H2 dislocation pattern was the most prevalent in both genders and in adults and elderly, we observed a different distribution of types in relation to men and women. All individuals with only anteriorization displacement (H1) were women (n=12) (Table 4).

**Table 4:** Distribution of women and men regarding the type of hyoid bone displacement (n=67)

Type	Women	Men
H1	12 (33.34)	0
H2	20 (55.55)	19 (61.29)
H3	1 (2.78)	0
H4	1 (2.78)	0
H5	0	1 (3.23)
H6	0	2 (6.45)
H7	2 (5.55)	9 (29.03)
Total	36 (100.00)	31 (100.00)

Results expressed in percentages (%).

H1: horizontal; H2: short vertical and long horizontal; H3: vertical and diagonal to top; H4: long vertical and short horizontal; H5: vertical; H6: diagonal; H7: short.

Regarding the distribution of individuals by sex and type of displacement (Table 4), the H7 pattern was more prevalent in men, *i.e*., approximately 82% of the individuals who had elevation and short anteriorization were men (n=9). Only men had H5 (n=1) and H6 (n=2) displacement patterns, and only women had H3 and H4 displacement types, both with one individual in each (Table 4).

## DISCUSSION

The displacement of the hyolaryngeal complex is an important component of the swallowing mechanism. This has a key role in controlling the opening of the upper esophageal sphincter, epiglottis tilt, and closure of the laryngeal vestibule.^(1,3,6,7,10,15,21)^ Seven displacement patterns were found in a group of 67 individuals with functional swallowing by means of descriptive analysis. The patterns were maintained in the individuals during swallowing of different consistencies.

Different studies since the 1990’s have analyzed, by means of objective examinations, the hyoid bone displacement during swallowing.^(3,5,9,11,13-16,19)^ The literature indicates that the vertical displacement of the hyoid bone contributes mainly to the closure of the laryngeal vestibule, while the anterior displacement contributes to the opening of the upper esophageal sphincter.^(3,21,22)^

Ishida et al.,^(23)^ in a study with healthy subjects by videofluoroscopy described that the hyoid bone moves upward and forward during swallowing. However, the upward displacement, at some specific times was small in relation to the anterior displacement. The amplitude of the displacement was highly variable, influenced by the consistency of the food and probably by the amplitude and vigor of the tongue propulsion movement at ejection. The authors proposed that events in the oral cavity (jaw and tongue movements) were the main regulators of the upward displacement of the hyoid bone during swallowing. The amplitude of anterior displacement of the hyolaryngeal complex was larger and less variable, which reinforces its important role in the opening of the upper esophageal sphincter.^(3,23)^

This study showed seven types of hyoid bone displacement patterns in a group of 67 healthy subjects, which demonstrated that hyoid bone movement is highly variable. The individual pattern did not vary its characteristics according to different food consistencies evaluated, it only appears to have modulated in amplitude and duration. Each individual in the study was able to promote a safe swallowing function within individual standards of normality. Thus, it is relevant to consider the presence of hyoid bone displacement variation even in healthy individuals.

Nagy et al. performed a study using videofluoroscopy of swallowing in 20 healthy subjects. The analysis revealed the influence of the consistency of the food bolus on the speed of anterior and superior hyoid movement. In other words, faster speeds of movement for thick consistency in nectar compared to thin and ultrafine liquid stimuli. The duration of the anteriorization and elevation of the hyoid bone was related to the time of laryngeal vestibule closure.^(21)^ Modulation of the amplitude of hyoid bone movements is a strategy for dealing with different volumes and consistencies of food bolus.

By varying the amplitude and duration of hyoid bone movement according to food volume, it is possible to maintain the upper esophageal sphincter opening long enough for bolus transit without impairing swallowing safety.^(3,21)^

Curtis et al. evaluated 65 subjects by means of videofluoroscopy of swallowing and showed that the superior and anterior displacements of the hyoid varied significantly according to the bolus volume. They also observed that gender significantly affected the superior hyoid displacement, but not its anterior displacement. Age significantly impacted the anterior displacement of the hyoid, however, it did not cause the superior displacement. Both superior and anterior displacements increased with increasing volume and were greater in men compared with women, and in adults compared to the elderly.^(3)^ These findings are similar to those of previous studies^(11,21-23)^ and to the results described here. Results demonstrated that hyoid displacement has variations in amplitude and duration according to food volume, and this can be influenced by age and sex in normal swallowing.

Different studies favor a highly variable nature of hyoid movement in the normal swallowing function by means of swallowing videofluoroscopy analysis.^(3,22,24)^ These findings allow the presence of seven different types of displacement in the described group. This occurs given the distinct elevation and anteriorization characteristics, according to multifactorial anatomical and physiological variables, presented in “normality”.^(3,4)^

Regarding the types of displacement, this study showed that approximately 60% of the sample had an H2 pattern. In other words, anteriorization predominated over elevation, and approximately 18% had an exclusively anterior movement (H1 pattern). Therefore, 78% of the subjects had predominant horizontal movement. This predominance reinforces some studies that pointed to its role in the opening of the upper esophageal sphincter, associated with the traction of the cricopharyngeal muscle. This finding allous the proper flow of the bolus and reducing the risks of laryngotracheal penetration and/or aspiration.^(3,5,13-16,22,25)^ However, it is not possible to conclude that horizontal movement is always more relevant than vertical movement in swallowing, mainly due to the fact that seven different types of movement were found.

This study also showed similar distribution of hyoid bone displacement patterns among adults and the elderly. However, despite the already known aspects of presbyphagia, it was observed that the elderly did not show a pattern of hyoid bone displacement distinct from the others. This leads us to suppose that the individuals maintain their swallowing pattern throughout life, despite possible adaptations and compensations that may occur with aging.

Regarding gender, the displacement patterns were different between men and women. Some studies pointed out important differences between genders in relation to hormones, anatomy, and growth of the structures responsible for phonation and swallowing.^(3,24)^ However, there are no studies proving the impact of these aspects on swallowing physiology. In the larynx, especially in relation to the thyroid cartilage, men have an angulation of about 90° and women about 120°, a difference that when associated with vocal fold length, impacts vocal physiology. It is possible to infer that these anatomical and size characteristics influence the diversification of the displacement pattern between men and women, according to their structural characteristics. Further detailed investigation of these aspects may contribute to increased knowledge in this area.

Rebrion et al. studied 24 individuals without swallowing disorders, evaluated by videofluoroscopy, and observed greater hyoid displacements in the vertical direction than in the horizontal direction. They stated that the vertical movement may be associated with a more appropriate laryngeal closure.^(26)^ Unlike the findings described in this study, Rebrion et al. reported that the anterior and superior displacement of the hyoid bone did not differ according to gender. However, the authors agreed that there was no variation in relation to the age of individuals.^(26)^

The hyoid bone characteristics, including its size and the relationship between its different structures, are highly heterogeneous in the population and they are closely linked to gender, height and weight of individuals.^(27)^ This bone morphology can influence both the displacement and biomechanics of the suprahyoid muscles.^(18)^ These aspects, associated with the findings of this study, reinforce the idea that swallowing is a complex process susceptible to the influence of several factors. The observations showed in this healthy population different types of hyoid bone displacement, with some patterns of higher prevalence and the maintenance of the displacement pattern for each individual. The swallowing of different consistencies, allows us to infer that these aspects are maintained throughout life, without prejudice or damage to the swallowing safety.

Regarding dysphagic individuals, Bingjie et al., in a study including 105 stroke patients and 100 normal adults, found an association between laryngeal penetration and aspiration and the vertical movement of the hyoid bone by videofluoroscopy.^(25)^ Kim et al. concluded, by means of videofluoroscopy of swallowing in 38 patients after stroke, that the horizontal movement of the hyoid was the most important factor in the discrimination of the aspiration event. These results were presented as late and sometimes reverse (from front to back).^(28)^ It is still unknown whether a certain displacement pattern contributes to the improvement or worsening of a swallowing disorder condition, or if some swallowing characteristic is more susceptible of becoming unsafe.^(4)^

In this study conducted from January 2014 to December 2016, 67 patients were identified with videofluoroscopic swallowing reports as normal or unchanged. This fact is justified because, in the studied service, the teams refer patients with suspected dysphagia to the outpatient and hospital levels for detailed investigation. The objective examination of swallowing, besides describing in detail changes in oropharyngeal and esophageal dynamics, can rule out the existence of dysphagia. It is natural that, after the objective evaluation, the existence of dysphagia can be confirmed or not.

All seven types of displacements found include variations of the movements described in the current literature, *i.e*., upward and/or forward. Strict inclusion and exclusion criteria were established to rule out any swallowing alteration. However, although the findings of the present study do not claim to represent a standard of normality, the reported here is still valuable for understanding the physiology of normal swallowing.

## CONCLUSION

Seven patterns of hyoid bone displacement were described during swallowing in adults and elderly with normal swallowing. The most frequent pattern of displacement was the H2 type, that is, short vertical and long horizontal displacement. There was with predominance of anteriorization over elevation. The H2 pattern was the one that presented the highest prevalence in both genders, both in adults and in elderlies. The percentage distribution of the types of hyoid displacement was different in men and women. The displacement pattern was maintained in all subjects during the swallowing of three different consistencies (thin liquid, pasty and solid).
